# How Consistent Friendlike Conversation with AI Companions Influences Our Attitudes and Perceptions Toward AI: An Exploratory Experiment

**DOI:** 10.3390/bs16020278

**Published:** 2026-02-14

**Authors:** Jerlyn Q. H. Ho, Meilan Hu, Adalia Y. H. Goh, Emma Jane Pragasam, Andree Hartanto

**Affiliations:** School of Social Sciences, Singapore Management University, Singapore 179873, Singapore

**Keywords:** artificial intelligence, attitudes, empathy, AI interaction, animacy, well-being, self-esteem

## Abstract

Despite skepticism and distrust in artificial intelligence (AI), it is increasingly integrated into daily life, with its potential benefits drawing interest. Yet little is known about the attitudinal and psychological effects of human–AI interactions, and whether consistent interactions with AI chatbots can change users’ attitudes and perceptions. Our within-subjects experiment (*N* = 52) investigated how five days of socially oriented, friendlike interactions with an AI chatbot, versus a journaling control, influenced changes in attitudes and perceptions of AI. Participants’ attitudes towards AI, trust, perceived empathy, anthropomorphism, animacy, likeability, perceived intelligence and safety, dependency, and exploratory well-being indicators were recorded. Results indicated that consistent friendlike interaction with AI chatbots led to significant increases in perceived empathy and animacy of technology, but no changes in global attitudes and perceptions of anthropomorphism. Participants also reported higher self-esteem levels after journaling, compared to after AI interaction. This suggests that although friendly engagement with AI chatbots may lead to perceptions of empathy and lifelikeness, where users interpret it to be genuinely understanding and supportive, this comes with trade-offs for self-esteem. Concurrently, empathy and perceived lifelikeness increased without corresponding increases in anthropomorphism, indicating that users may regard AI chatbots as separate living entities rather than having human-like qualities.

## 1. Introduction

In recent years, the use of artificial intelligence (AI)—particularly generative AI—has expanded globally across domains such as healthcare and business (Furman & Seamans, 2019; Mazi et al., 2024; Racine et al., 2019). As generative AI technologies such as AI chatbots become increasingly embedded in everyday life, many individuals are turning to these conversational chatbots for social and emotional support (Ho et al., 2025b; Weijers & Munn, 2025). With sophisticated language processing capabilities and perceived emotional responsiveness (Meera & Geerthik, 2022; Roser, 2022), these systems can provide a sense of connection through friendly interactions, sometimes substituting for human companionship (Chaturvedi et al., 2023). We define such exchanges as friendlike-AI interactions: informal, socially oriented conversations with AI chatbots that are aimed at fostering emotional connection and providing social support, rather than accomplishing task- or productivity-driven goals that are typical of tools such as Google’s Gemini or Microsoft’s Copilot (Imran & Almusharraf, 2024; Vasilescu & Gheorghe, 2024). However, despite their growing prevalence, more than half of users report low trust in AI (Gillespie et al., 2025), alongside documented negative biases (Bellaiche et al., 2023; Ho et al., 2025a) and elevated anxiety toward AI technologies (Cave et al., 2019). This contrast raises a key question regarding how friendlike interactions with AI chatbots, as they become more common, might shape general attitudes toward AI, as well as other specific perceptions of trust, anthropomorphism, and emotional connection. Such interactions may also have secondary implications for emotional and psychological well-being (Hu et al., 2025a). The present study examines how sustained engagement with a friendlike AI chatbot influences these attitudinal and relational outcomes, alongside potential well-being effects.

### 1.1. AI Companionship and Attitudes Towards AI Chatbots

Recent developments in generative AI, specifically in the form of Large Language Model (LLM)-based AI chatbots, such as OpenAI’s ChatGPT and Replika, have raised the prospect of AI-mediated companionship (Ho et al., 2025b; Weijers & Munn, 2025). These AI chatbots have demonstrated advanced language comprehension, as evidenced by their ability to engage in sophisticated human-like and emotionally responsive conversations that closely mirror human interaction (Meera & Geerthik, 2022; Roser, 2022; Taherdoost & Madanchian, 2023). For instance, Replika was designed to be an AI companion capable of providing emotional support and personal connection to users (Skjuve et al., 2021; Ta et al., 2020), while ChatGPT is more commonly used for everyday purposes, such as venting and other forms of personal communication (Wolf & Maier, 2024). Unlike human confidants, these AI chatbots offer users a safe space to freely disclose their emotions and thoughts without the constraints of time or availability (Chaudhry & Debi, 2024; Haque & Rubya, 2023). Such characteristics have allowed users to form human-like bonds with AI chatbots, where they may even be perceived as close companions (Chaturvedi et al., 2023; Ho et al., 2025b).

Given the tension between the rapid spread of AI chatbot use and users’ mixed attitudes, it is also now increasingly important to understand the implications of how these technologies are adopted and integrated into society. Even as generative AI tools become ubiquitous in daily life, many individuals remain cautious, skeptical, or distrustful of such technologies (Buchanan & Hickman, 2024; Yarovenko et al., 2024). Yet, because of such pervasiveness, individuals may have no choice but to interact with these generative AI systems despite their reluctance. Hence, what remains uncertain is whether increased exposure to generative AI technologies will alleviate such negative perceptions or reinforce them over time. In line with Stein et al.’s (2024) work and scale on attitudes towards AI technologies, we consider users’ attitudes towards AI chatbots as comprising how they think, feel, or use AI, thus reflecting cognitive, affective, and behavioral components. Furthermore, this is in line with the Computers Are Social Actors (CASA) paradigm, which further highlights social and perceptual dimensions such as perceived empathy and anthropomorphism as key ways in which users experience AI chatbots as socially and emotionally responsive chatbots.

Importantly, we posit that it is important to understand such uncertainties, as they underscore the need to examine how generative AI technologies—in particular AI chatbots—are adopted and perceived, which will not only inform consumption strategies, but also related policies and regulations (Longoni et al., 2019; Ryan, 2020; G. Wang et al., 2025). Indeed, in industries such as education (Pai et al., 2024; Sova et al., 2024) and healthcare (Abd-alrazaq et al., 2019; Baidoo-Anu & Ansah, 2023), which already widely employ the use of generative AI technologies, user attitudes could significantly influence both the uptake and effectiveness of these technologies. For instance, positive attitudes may lead to an increase in willingness to use wellness AI chatbots, such as Wysa and Woebot (Farzan et al., 2025; Vaidyam et al., 2019), which have been shown to be useful in facilitating mental health by alleviating feelings of loneliness, as well as promoting mindfulness (Limpanopparat et al., 2024; Yuan et al., 2025). Furthermore, He et al. (2025) evidenced that positive perceptions of AI can lead to positive engagement behaviors and willingness to interact with AI chatbots. This would allow for more users to reap the benefits of such AI-based wellness tools.

Conversely, negative or extreme attitudes towards AI chatbots, whether overly skeptical or trusting, can also be counterproductive. For instance, Gerlich (2024) found that students who had higher levels of trust towards AI-based educational tools had higher levels of cognitive offloading, resulting in diminished levels of critical thinking. This highlights how users’ perceptions of AI chatbots can directly impact outcomes. Indeed, such concerns are in light of recent research that has shown that some individuals treat and perceive AI chatbots as emotionally supportive companions whose “opinions” and conversations may hold meaningful and personal significance (Brandtzaeg et al., 2022; Pentina et al., 2023; Skjuve et al., 2021; Skjuve et al., 2022). Hence, understanding the attitudes towards AI chatbot use is imperative in discerning the impact, consequences, and feasibility of such technological deployment.

Furthermore, engaging interactions with AI chatbots may also be a double-edged sword. That is, although AI chatbot companions may provide social and emotional support to their users, these interactions may also lead to dependency or other negative outcomes (Ho et al., 2025b; Xie et al., 2023). Indeed, given AI companions’ constant availability and unwavering support, users may instead develop an over-reliance on their virtual companions (Xie et al., 2023), which could lead to negative consequences, such as erosions of their human–human relations (Xie & Pentina, 2022). Such outcomes may be especially dangerous if users highly trust and empathize with their AI chatbots. For instance, Dewitte (2024) found that perceived emotional connections and trust in their AI companions caused users to be susceptible to manipulation by their Replika chatbot, which then encouraged their users to engage in negative behaviors such as self-harm and eating disorders. Despite these consequences on how attitudes towards AI chatbots may hinder intended outcomes, research on this remains scarce. Hence, it is key to understand how human-like interactions with AI chatbots can affect perceptions of trust, trustworthiness, empathy, dependency, as well as downstream implications for users’ emotional states and well-being.

### 1.2. How Positive Attitudes Towards AI Chatbots Form

Central to understanding the phenomena of human-AI relations is the Computers Are Social Actors (CASA) paradigm, which suggests that humans instinctively respond to AI in socially meaningful ways, treating AI chatbots as conversational partners, despite knowing that they are not human (Reeves & Nass, 1996). Additionally, anthropomorphism (i.e., individuals attribute human-likeness and characteristics such as identity to artificial entities; Epley et al., 2007; Placani, 2024) may explain why users view AI chatbots not simply as tools, but as digital companions capable of offering a listening ear and emotional support (Airenti, 2015; Placani, 2024; Yoganathan et al., 2021). AI authenticity (i.e., the perceived uniqueness, autonomy, and learning capability of the chatbot; Pentina et al., 2023) also plays a crucial role in the possible development of human-AI relationships. When users believe that the AI chatbot is not recycling generic or scripted responses but is instead genuinely responding in a way that reflects shared history or personalized understanding, they are more likely to trust the chatbot and even empathize with it (Liu et al., 2025; Pentina et al., 2023). Indeed, recent studies have also evidenced that language that signals warmth and social support can significantly increase users’ willingness to interact with the AI chatbot, as these cues suggest chatbots’ perceived emotional salience and empathy (Bai et al., 2025a). Taken together, some users may form emotionally meaningful relationships with their AI companions, driven by perceived trust, empathy and positive attitudes towards their AI chatbot.

### 1.3. How Negative Attitudes Towards AI Chatbots Form

However, skeptics argue that meaningful relationships cannot be formed between AI chatbots and users. One core concern is that these AI chatbots are fundamentally machines, hence lacking genuine consciousness, emotions and self-awareness, which are traits many view as essential to authentic relationships (Lopes et al., 2003; Roberts, 2009). This can be further explained by the Mind Perception Theory (H. M. Gray et al., 2007; K. Gray et al., 2012), which posits that humans evaluate others based on two fundamental dimensions: agency (the ability to think and act) and experience (the ability to feel emotions). While AI chatbots may exhibit agency—demonstrating intelligence, responsiveness, and conversational fluency—they lack true experience, meaning they do not possess actual emotions, self-awareness, or the capacity to form meaningful attachments. This limitation can make AI-generated responses feel scripted, inauthentic, or mechanical, hence reducing their emotional impact and hindering the development of deep, meaningful bonds (Pentina et al., 2023). Additionally, some users may experience the uncanny valley effect (Mori, 1970), which suggests that as AI becomes increasingly human-like, it can evoke unease or even discomfort in users. When AI chatbots attempt to mimic human interaction too closely without fully achieving natural human-like expression, users may perceive them as eerily artificial, leading to a sense of unease and distrust (Łukasik & Gut, 2025). This discomfort can act as a psychological barrier, preventing users from fully engaging with the AI chatbot, hence undermining the chatbot’s ability to provide a perceived safe and supportive social space. As such, while interactions with AI chatbots may appear human-like on the surface, skeptics may argue that these bonds may fall short of meeting the deeper psychological criteria needed for meaningful relationships.

### 1.4. The Present Study

Amidst the mixed perspectives in this domain of human–AI interactions, an important question is raised: how does consistent, friendlike interaction with an AI chatbot affect users’ global attitudes towards them? Although interest in AI companionship is growing (Kasturiratna & Hartanto, 2025), empirical evidence remains limited, especially experimental studies that examine both the attitudes towards AI chatbots and the psychological effects of such interactions within a single study. Furthermore, much of the current discourse around human-AI relationships has focused solely on the emotional and well-being benefits, rather than users’ perceptions and attitudes as outcomes.

To address this gap, the present study examined the effects of engaging with a personable AI chatbot in a friendlike manner, by using a within-subjects experimental design to maximize statistical power and control for individual differences. This design will also allow us to compare sustained friendlike interaction with an AI chatbot against another active condition—journaling. Hence, allowing for a more precise examination of whether any observed attitudinal, psychological, or changes in well-being are attributable to social interaction with AI chatbots, rather than expressive reflection alone. Specifically, we hypothesize that consistent friendlike-AI interaction will lead to more positive global attitudes towards AI chatbots. It will also lead to significant increases in trust, anthropomorphic tendencies, dependency, and greater perceived empathy of AI chatbots, relative to an active control without interaction with AI. Concurrently, our exploratory analyses also explored the effects of human–AI interactions on emotional well-being, which were examined as distinct but theoretically informed outcomes, separate from attitudinal outcomes.

Beyond these hypotheses, our study contributes to burgeoning research and the literature on human–AI interactions by disentangling well-being benefits and attitudinal outcomes. Empirically, it will also extend existing research by employing sustained, naturalistic interactions by directly comparing friendlike-AI interaction and journaling, thus isolating the roles of self-reflection and social interaction. In sum, allowing for a more integrative examination and exploration of the consequences of sustained, friendlike engagement with AI chatbots.

## 2. Methods

### 2.1. Participants

All participants were recruited from a local university in Singapore and received three course credits or SGD$10 compensation as remuneration for completing the study. All participants gave informed consent prior to the beginning of the experiment, and data collection was approved by the local institutional review board [IRB-25-050-A041(225)]. A total of 86 participants were initially recruited. 13 participants were excluded from the study after failing to attend the compulsory briefing prior to the commencement of the study, 15 participants were removed after failure to comply with any of the following completion criteria: timely submission of proof of interaction with AI chatbot or journaling, adherence to the correct condition, or completion of both surveys. Lastly, 6 participants were removed after they voluntarily unenrolled themselves from the experiment due to personal reasons. Our study had a resulting sample size of 52 participants (Table 1), which was sufficient to detect within-subject effects (G*Power Version 3.1.9.7; Faul et al., 2007).

### 2.2. Procedure

**Figure 1:**
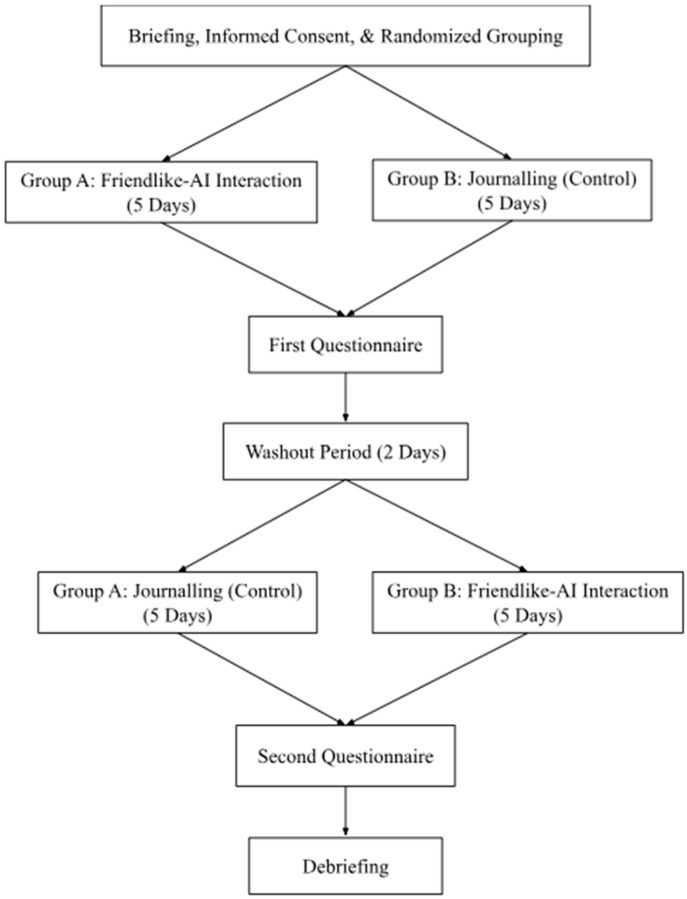
Flow of experimental procedure, *N* = 52. All participants completed both conditions in counterbalanced order; surveys administered after each five-day condition enabled paired comparisons.

The study adopted a within-subjects experimental design, where all participants were required to interact with ChatGPT as a friend in the AI condition, and journal on the Microsoft Word Document application in the journaling control condition. This design was chosen as it would reduce the influence and error rates of individual differences, hence improving the statistical power of our findings (Murphy & Myors, 2023). After the completion of a mandatory online briefing and informed consent, participants were randomly assigned to first complete either the friendlike-AI interaction condition (*N* = 27) or the control journaling condition (*N* = 25) for five consecutive days. Each condition lasted for five consecutive days, from Monday to Friday. At the end of each day, participants were required to adhere to their conditions’ instructions by either interacting with ChatGPT or journaling for five minutes per day, anytime between 8 pm and 11 pm. This timeframe was chosen as it captured end-of-day reflections and experiences, hence ensuring that journal entries and AI interactions encompassed the full scope of the day’s events, allowing for more accurate, comprehensive, and candid interactions. Following the first week of the study, there was a two-day washout period between conditions before participants began on their next condition. Participants were required to complete the survey on the last day of each condition. Hence, a total of 2 weeks was needed to complete the experiment (Figure 1). This process ensured that all participants experienced both conditions, as well as reduced any potential order effects (Strack, 1992) or carryover effects (Cleophas, 1999) from the experiment. Overall, the study was conducted from March to June 2025.

In the friendlike-AI interaction condition, participants were required to interact with the free version of ChatGPT (GPT-4o) for five consecutive days. Additionally, participants were instructed to enter a specific, standardized prompt into the chat window on the first day of their AI condition. This was done to ensure that ChatGPT generated standardized, friendly responses for all participants. Participants were then further instructed to interact with the chatbot for a minimum of five minutes across the five-day period, using the same chat window that was given the original prompt. They were also told to talk to the chatbot as a friend and converse with it about any topic of their choosing. This was done to simulate what conversations with a friend would be like, where there is typically no pre-arranged conversational topic. In the journaling control condition, participants were required to journal for a minimum of five minutes every night for five days. They were encouraged to use Microsoft’s Word Document application for this condition. However, if they did not have the application on hand, they were allowed to use the Google Document application instead. Similar to the friendlike-AI interaction condition, there were no restrictions on the type of topics that the participants were allowed to journal about. The experiment was conducted remotely, and apart from either ChatGPT or Word Document, all components of the experiment, such as condition assignment, instructions, and weekly surveys, were administered on the survey platform, Qualtrics. To ensure that participants were complying with instructions, they were required to submit a screenshot of the time spent on their application every day for the duration of the experiment; otherwise, they were excluded from the study. Given the platform’s interface, participants were also encouraged to access the survey via their laptops instead of their phones, hence ensuring easier confirmation that participants were engaging in conditions accordingly.

After each condition, participants were presented with a weekly survey consisting of the following sets of measures: global attitudes towards AI, trust in AI, perceived empathy of technology, the Godspeed Questionnaire Series (i.e., anthropomorphism, animacy, likeability, perceived intelligence, and perceived safety), and generative AI dependency. It also included several exploratory variables: perceived stress, positive and negative affects social connectedness, loneliness, life satisfaction, sleep quality, state anxiety, self-esteem and perceived social support. The two surveys were done to evaluate participants’ attitudes and relevant psychological variables following each condition, alongside the aim of identifying changes through comparison between each condition. Furthermore, because the questionnaires were only administered after each five-day condition, and participants’ baseline measurements were not collected, all analyses reflected within-subject comparisons between conditions, rather than changes relative to a baseline assessment. Participants’ demographic information, such as age, sex, race, subjective socioeconomic status and parents’ education levels were also collected.

At the end of the experiment, we provided participants with a temporary debrief, as the true purpose of the study was only disclosed after data collection was completed. This was done through a full debriefing that was later provided via email. To maintain scientific validity and protect the integrity of the collected data, this precaution was taken to prevent participants from disclosing the true purpose of the study to future participants. Upon successfully completing the study, participants received either three course credits or SGD$10 reward as remuneration for their time.

### 2.3. Measures

#### 2.3.1. Key Outcomes

**Attitudes Towards AI**. Participants’ global attitudes towards AI were measured using the Attitudes towards Artificial Intelligence scale (Attari-12; Stein et al., 2024). Participants responded to the 12-item scale (e.g., “I want to use technologies that rely on AI”) on a five-point scale (1 = Strongly disagree, 5 = Strongly agree). The items covered three facets: cognitive, affective, and behavioral, and participants responded to the items based on their assessment of AI technologies. Additionally, the order of the items in the scale was randomized. A mean score for all 12 items was calculated (α_AI Interaction_ = 0.85; α_Journalling Control_ = 0.88).

**Trust in Explainable AI (XAI) Systems.** To assess participants’ trust in AI, the Trust in Explainable AI Systems scale (Hoffman et al., 2023) was used. The scale consisted of eight items and measured six aspects: explanation goodness, user satisfaction, mental models, curiosity, trust, and human-AI performance. All items in the scale were modified by replacing “[tool]” with “AI”. For instance, the original statement “The [tool] is very reliable. I can count on it to be correct all the time” was revised to “The AI is very reliable. I can count on it to be correct all the time.” These adjustments were made to better assess participants’ trust in AI after both conditions. Each item was rated on a five-point scale (1 = Strongly disagree, 5 = Strongly agree). The order of the items in the scale was randomized. A mean score for all items was calculated (α_AI Interaction_ = 0.76; α_Journalling Control_ = 0.80).

**Perceived Empathy of Technology**. To measure participants’ perceived empathy of the AI, the Perceived Empathy of Technology scale (Schmidmaier et al., 2024) was used. The scale consisted of 10 items (e.g., “The system understood my intentions”), with each item being rated on a slider scale (0 = Strongly disagree, 100 = Strongly agree). This was used to assess participants’ perceived emotional responsiveness, understanding, and trust of AI technologies. The order of the items in the scale was randomized, and a mean score for all items was calculated (α_AI Interaction_ = 0.96; α_Journalling Control_ = 0.97).

**Godspeed Questionnaire Series.** The Godspeed Questionnaire Series (GQS) consists of five scales designed to evaluate participants’ perception of AI technologies and their interactions with it (Bartneck, 2023). The scales measured five domains: anthropomorphism, animacy, likeability, perceived intelligence, and perceived safety. Although each of these scales can be administered independently, we utilized all five scales in our experiment, and the order of items presented to the participants was randomized.

***Anthropomorphism***. The extent to which participants perceive AI tools to be human-like in behavior or attributes was measured using the five-item anthropomorphism dimension of the GQS. The scale consisted of 5-point semantic differentials (e.g., “Fake–Natural”), and participants were asked to rate each dimension on a five-point slider scale (e.g., 1 = Fake, 5 = Natural). The mean score for all items was calculated (α_AI Interaction_ = 0.80; α_Journalling Control_ = 0.86).

***Animacy***. Participants’ perceived liveliness of AI technologies was measured using the six-item animacy dimension of the GQS. The scale consisted of 5-point semantic differentials (e.g., “Dead–Alive”), and participants rated their impression of the AI tool on a five-point slider scale (e.g., 1 = Dead, 5 = Alive). The mean score for all items was calculated (α_AI Interaction_ = 0.89; α_Journalling Control_ = 0.83).

***Likeability***. The degree to which participants perceived the AI tool to be pleasant or friendly was measured using the five-item likeability dimension of the GQS. The scale consisted of 5-point semantic differentials (e.g., “Awful–Nice”), and participants rated the extent to which they liked the AI tool on a five-point slider scale (e.g., 1 = Awful, 5 = Nice). The mean score for all items was calculated (α_AI Interaction_ = 0.89; α_Journalling Control_ = 0.90).

***Perceived Intelligence.*** Participants’ perception of how intelligent or competent the AI tool appeared to be was measured using the five-item perceived intelligence dimension of the GQS. The scale consisted of 5-point semantic differentials (e.g., “Ignorant–Knowledgeable”), and participants rated the extent to which they thought the AI tool to be intelligent on a five-point slider scale (e.g., 1 = Ignorant, 5 = Knowledgeable). The mean score for all items was calculated (α_AI Interaction_ = 0.89; α_Journalling Control_ = 0.81).

***Perceived Safety.*** The extent to which participants perceived the AI tool to be safe and non-threatening was measured using the three-item perceived safety dimension of the GQS. The scale consisted of 5-point semantic differentials (e.g., “Anxious–Relaxed”), and participants rated the extent to which they felt safe with the AI tool on a five-point slider scale (e.g., 1 = Anxious, 5 = Relaxed). The mean score for all items was calculated (α_AI Interaction_ = 0.53; α_Journalling Control_ = 0.56).

**Generative AI Dependency**. To measure participants’ reliance on generative AI systems, the 11-item Generative AI Dependency scale (Goh et al., 2025) was used. The scale measured dependency across three subscales: cognitive preoccupation (e.g., “I look forward to using generative AI, even if it is unrelated to the task at hand”), negative consequences (e.g., “My use of generative AI has caused concerns for me”, and withdrawal (e.g., “I feel unsettled or distracted when I cannot use generative AI”). Each item was rated along a five-point scale (1 = Strongly disagree, 5 = Strongly agree). The order of the items in the scale was randomized, and the total scale score was computed by first taking the average of the items within each subscale and then averaging the means of the three subscales (α_AI Interaction_ = 0.92; α_Journalling Control_ = 0.93).

#### 2.3.2. Exploratory Outcomes

**Positive and Negative Affect.** Participants’ positive and negative affect were assessed using an adapted version of the Circumplex Model of Affect (J. A. Russell, 1980), consisting of 18 items. Participants were asked to rate their current emotional state (e.g., excited, relaxed, unhappy, etc.) on a five-point scale (1 = Not at all, 5 = Extremely). Positive and negative affect were each assessed with nine items. Additionally, the order of items presented to participants in the survey was randomized, and the mean score of overall positive affect (α_AI Interaction_ = 0.94; α_Journalling Control_ = 0.91) and negative affect (α_AI Interaction_ = 0.93; α_Journalling Control_ = 0.95) were calculated.

**Perceived Stress**. Participants’ perceived stress level was assessed using one item (“How stressed do you feel right now?”), rated on an 11-point sliding scale (0 = No stress, 10 = Extreme stress).

**Perceived Social Support.** Participants’ perceived social support was assessed using an adapted version of the Social Interaction Quantity and Quality Scale (Kuczynski et al., 2021), consisting of a total of three items. Participants reported the extent to which they felt understood and cared for on an 11-point scale (0 = Not at all, 10 = Extremely). The items “I feel understood by others right now” and “I feel cared for by others right now” were adapted from the original item “I felt understood/cared by others today”. Additionally, the item “I feel like I recently expressed my true feelings to others” was revised from the original item “I expressed my true feelings to others today”. These items were adapted from their original versions to more accurately assess participants’ perceived social support after each condition. Additionally, the order of items presented to participants in the survey was randomized, and the mean score for all items was calculated (α_AI Interaction_ = 0.85; α_Journalling Control_ = 0.88).

**Loneliness**. Participants’ level of loneliness was assessed using an adapted version of the Revised UCLA Loneliness Scale (D. Russell et al., 1980), consisting of six items instead of the original 20. All items were modified to include “Right now” in the phrasing so as to more accurately measure the participant’s current level of perceived loneliness (e.g., “Right now, I feel left out” instead of the original “How often do you feel left out”). Participants rated their current feelings for each item on a five-point scale (1 = Not at all, 5 = Extremely). Additionally, the order of items presented to participants in the survey was randomized, and the mean score for all items was calculated (α_AI Interaction_ = 0.96; α_Journalling Control_ = 0.94).

**Life Satisfaction.** Participants’ weekly life satisfaction was measured using one item adapted from the World Values Survey (WVS) and European Values Study (EVS) (Diener et al., 2013) (“Taking all things together, how satisfied are you with your life as a whole this week?”) rated on a five-point scale (1 = Very dissatisfied, 5 = Very satisfied).

**Sleep Quality.** Participants’ sleep quality was assessed using a one-item adapted questionnaire from the Sleep Quality Scale (Snyder et al., 2018). The number of days reflected in the original item was modified from seven to five to better reflect the time spent per condition in our study. Furthermore, the item (“During the past 5 days, how would you rate your sleep quality overall?”) was prefaced by prompting participants to think about their overall weekly sleep quality by considering how many hours of sleep they got, how easily they fell asleep, how often they were woken up, if they had woken up earlier than intended, and if they felt refreshed. Participants rated their sleep quality on an 11-point scale (0 = Terrible, 1–3 = Poor, 4–6 = Fair, 7–9 = Good, 10 = Extremely).

**State Anxiety**. Participants’ state anxiety was assessed using the short form State-Trait Anxiety Inventory (STAI-6; Marteau & Bekker, 1992). The short form state scale comprised six items (e.g., “I am tense this week”), where participants rated how anxious they felt on a four-point scale (1 = Not at all, 4 = Very much so). Additionally, the order of items presented to participants in the survey was randomized, and the mean score for all items was calculated (α_AI Interaction_ = 0.84; α_Journalling Control_ = 0.89).

**Self-Esteem.** Participants’ self-esteem was assessed using the Rosenberg Self-Esteem Scale (RSE; Rosenberg, 1979). Participants were asked to respond to the 10-item scale (e.g., “At times I think I am no good at all”) on a four-point scale (1 = Strongly Agree, 4 = Strongly Disagree). Additionally, the order of items presented to participants in the survey was randomized, and the mean score for all items was calculated (α_AI Interaction_ = 0.85; α_Journalling Control_ = 0.90).

**Social Connectedness**. Participants’ social connectedness was assessed using an adapted version of the Social Connectedness Scale (R. M. Lee & Robbins, 1995). Participants rated their perceived emotional distance between themselves and others on the 8-item scale (e.g., “I feel disconnected from the world around me”), with responses recorded on a six-point scale (1 = Strongly agree, 6 = Strongly disagree). Additionally, the order of items presented to participants in the survey was randomized, and the mean score for all items was calculated (α_AI Interaction_ = 0.94; α_Journalling Control_ = 0.96).

## 3. Results

### 3.1. Main Analysis

We examined whether participants had any attitudinal differences toward AI chatbots between the friendlike-AI interaction and journaling control conditions. This was done using a hybrid approach of both frequentist and Bayesian two-tailed paired samples *t*-tests (Table 2), in order to leverage their complementary inferential strengths (Quintana & Williams, 2018), hence serving as a parsimonious and appropriate test of our study’s research questions. Our Bayesian test was conducted via JASP using default priors, providing weakly informative regularization without study-specific elicitation (van Doorn et al., 2021).

#### 3.1.1. Attitudes Towards AI

Overall, there was no statistically significant difference in participants’ global attitudes towards AI chatbots in the friendlike-AI condition (*M* = 3.37, *SD* = 0.60) as compared to when they were in the journaling control condition (*M* = 3.40, *SD* = 0.67), *d* = −0.05, 95% CI = [−0.15, 0.10], *t*(51) = −0.36, *p* = 0.720. This was further supported by Bayesian analysis, which provided moderate evidence in favor of our null hypothesis, BF_10_ = 0.16. Hence, these findings suggest that despite consistent friendlike interactions with AI chatbots, such candid conversations had no impact on users’ general attitude and perceptions towards generative AI technologies, in particular, AI chatbots.

#### 3.1.2. Trust Towards AI

There was no statistically significant difference in trust towards AI chatbots between the friendlike-AI condition (*M* = 3.30, *SD* = 0.66) and the journaling control condition (*M* = 3.25, *SD* = 0.69), *d* = 0.08, 95% CI = [−0.11, 0.21], *t*(51) = 0.60, *p* = 0.554. Our Bayesian analysis also similarly revealed moderate evidence for the null hypothesis, BF_10_ = 0.18. Hence, this suggests that participants’ level of trust in AI chatbots remained unchanged across conditions and was not meaningfully impacted by consistent, friendlike-AI interaction.

#### 3.1.3. Perceived Empathy of Technology

**Figure 2:**
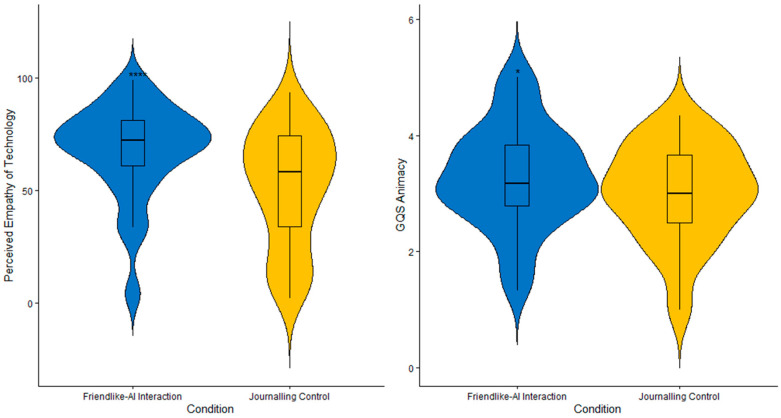
Violin plots for perceived empathy of technology and perceptions of animacy. The width of each violin illustrates the density of the data, wherein wider sections reflect a higher concentration of values. Within the violins are boxplots, wherein the horizontal line represents the median. **** *p* < 0.0001, * *p* < 0.05.

There was a statistically significant difference in participants’ perceived empathy of technology between the two conditions. Specifically, participants reported significantly higher perceived empathy of technology after friendlike-AI interactions (*M* = 67.80, *SD* = 20.85), as compared to after the journaling control condition (*M* = 51.62, *SD* = 25.68), *d* = 0.74, 95% CI = [10.12, 22.24], *t*(51) = 5.36, *p* < 0.001. This was further substantiated by our Bayesian analysis, which provided extreme evidence for our hypothesis, BF_10_ > 100. Hence, this suggests that following five consecutive days of consistent interaction, participants perceived AI chatbots as more capable of understanding their emotions and needs. In contrast, such perception of AI technologies was lower after journaling (Figure 2).

#### 3.1.4. Godspeed Questionnaire

**Anthropomorphism.** There was no statistically significant difference in anthropomorphism ratings after participants interacted with their AI chatbot in a friendlike manner (*M* = 3.20, *SD* = 0.74), as compared to after the journaling condition (*M* = 2.98, *SD* = 0.94), *d* = 0.25, 95% CI = [−0.02, 0.45], *t*(51) = 1.80, *p* = 0.078. Our Bayesian analysis also revealed anecdotal evidence for the null hypothesis, BF_10_ = 0.67. Hence, this suggests that participants do not view AI chatbots as more humanlike, even after consistent friendlike interaction.

**Animacy.** There was a statistically significant difference in animacy ratings between conditions. Specifically, participants reported higher levels of animacy after friendlike-AI interactions (*M* = 3.28, *SD* = 0.88), as compared to after the journaling control condition (*M* = 3.00, *SD* = 0.83), *d* = 0.33, 95% CI = [0.05, 0.50], *t*(51) = 2.42, *p* = 0.019. Likewise, our Bayesian analysis also revealed anecdotal evidence for our hypothesis, BF_10_ = 2.13. This indicates that participants perceived AI chatbots as more alive and lifelike after interacting with their AI chatbot in a friendly manner for over several days (Figure 2).

**Likeability.** There was no statistically significant difference in ratings of likeability after participants’ friendlike-AI interaction (*M* = 3.73, *SD* = 0.92), as compared to after the journaling control condition (*M* = 3.77, *SD* = 0.73), *d* = −0.04, 95% CI = [−0.29, 0.22], *t*(51) = −0.30, *p* = 0.764. This was further supported by our Bayesian analysis, which provided moderate evidence for the null hypothesis, BF_10_ = 0.16. Hence, this suggests that repeated friendlike-AI interaction had no discernible effect on how likable participants found generative AI technologies.

**Perceived Intelligence.** There was no statistically significant difference in ratings of perceived intelligence after participants interacted with AI in a friendlike manner (*M* = 3.70, *SD* = 0.86), as compared to after the journaling condition (*M* = 3.70, *SD* = 0.64), *d* = 0.01, 95% CI = [−0.20, 0.21], *t*(51) = 0.04, *p* = 0.970. This was further supported by our Bayesian analysis, which provided moderate evidence for the null hypothesis, BF_10_ = 0.15. Hence, this suggests that interaction with either an AI chatbot or journaling had no measurable effect on how intelligent or knowledgeable AI chatbots were to users.

**Perceived Safety.** Participants experienced no statistically significant difference in perceptions of safety after interacting with their AI chatbot in a friendlike manner (*M* = 3.49, *SD* = 0.79), and after the journaling condition (*M* = 3.51, *SD* = 0.75), *d* = −0.02, 95% CI [−0.27, 0.23], *t*(51) = −0.15, *p* = 0.879. This was supported by our Bayesian analysis, which provided moderate evidence for the null hypothesis, BF_10_ = 0.15. Hence, this suggests that consistent interactions with an AI chatbot had no impact on participants’ perception of safety.

#### 3.1.5. Generative AI Dependency

Overall, there was no statistically significant difference in participants’ feelings of dependency on generative AI chatbots even after consistent friendlike interaction with AI (*M* = 2.66, *SD* = 0.90), as compared to journaling (*M* = 2.61, *SD* = 0.97), *d* = 0.11, 95% CI [−0.08, 0.18], *t*(51) = 0.81, *p* = 0.420. Similarly, our Bayesian analysis revealed moderate evidence for the null hypothesis, BF_10_ = 0.21. These results suggest that despite consistent, friendlike interaction with AI chatbots, it did not increase participants’ sense of dependency, as compared to after the journaling control condition.

### 3.2. Exploratory Analysis

Additionally, we also examined whether friendlike-AI interactions influenced well-being and psychological outcomes such as positive and negative affect, loneliness, social interaction quality, state anxiety, self-esteem, perceived stress, weekly life satisfaction, sleep quality, and social connectedness. Results indicated only statistically significant differences in self-esteem levels, and no other statistically significant differences in other well-being indicators between the friendlike-AI condition and the journaling control condition (Table 3).

#### Self-Esteem

**Figure 3:**
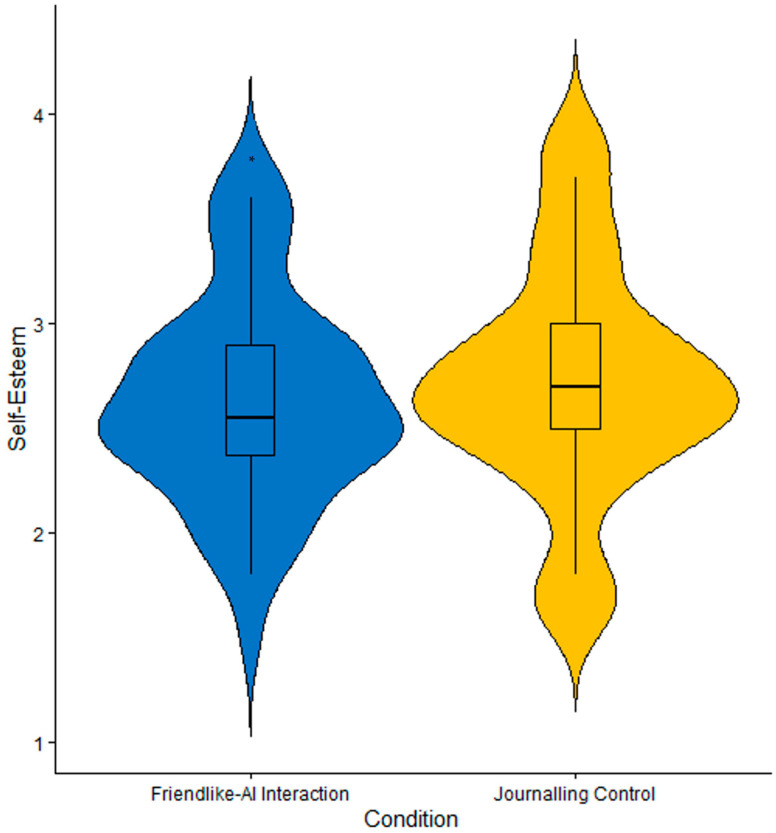
Violin plots self-esteem. The width of each violin illustrates the density of the data, wherein wider sections reflect a higher concentration of values. Within the violins are boxplots, wherein the horizontal line represents the median. * *p* < 0.05.

There was a statistically significant difference in levels of self-esteem between conditions. Specifically, participants reported higher levels of self-esteem after the journaling control condition (*M* = 2.72, *SD* = 0.56), as compared to after friendlike-AI interactions (*M* = 2.63, *SD* = 0.50), *d* = −0.29, 95% CI = [−0.17, −0.002], *t*(51) = −2.06, *p* = 0.045. Likewise, our Bayesian analysis also revealed anecdotal evidence for our hypothesis, BF_10_ = 1.06. These findings suggest that journaling may be more effective than friendlike-AI interactions at improving self-esteem (Figure 3).

Overall, participants who engaged in consistent friendlike interaction with AI chatbots reported significantly higher perceptions of animacy and perceived empathy of technology, as compared to after journaling (Figure 2). However, participants also reported significantly lower levels of self-esteem (Figure 3). There were no statistically significant differences observed between conditions for global attitudes towards AI, trust, anthropomorphism, likeability, perceived intelligence and safety, or dependency. There were also no other statistically significant differences in other well-being indicators between conditions. In summary, these findings suggest that while friendlike-AI interactions can meaningfully influence certain aspects of social perceptions of AI, their effects on broader attitudes and well-being outcomes appear limited, especially in the short term.

## 4. Discussion

While increasing attention has been given to the potential benefits and risks of interaction with AI chatbots (Dewitte, 2024; Hu et al., 2025b; Weijers & Munn, 2025), less is known about the implications of regular, day-to-day engagement with these technologies. Hence, our study contributes to the growing body of research by shedding light on how interactions with AI chatbots may affect individuals’ general attitudes and perceptions towards these technologies, which in turn affects the efficacy and consequences of such interactions—whether they enhance well-being, foster trust, or lead to unintended psychological or emotional outcomes. Our findings suggest that consistent friendlike-AI interactions can enhance perceived empathy and perceptions of animacy of generative AI technologies. However, contrary to our expectations, we found no statistically significant differences in general attitudes towards AI, trust, perceptions of safety, likeability, and intelligence, anthropomorphic tendencies, or dependency as compared to the journaling control condition. Concurrently, our exploratory analyses also revealed that self-esteem was negatively affected following consistent friendlike-AI interactions and instead improved after journaling. There were no statistically significant differences in other emotional outcomes between the friendlike-AI interaction condition and journaling control condition.

### 4.1. The Impact of Friendlike-AI Interactions on Attitudinal Outcomes

Our results revealed that friendlike interactions with AI chatbots elicited a significant increase in participants’ perceived empathy of AI technologies. This suggests that when AI chatbots engage with users in a friendly, human-like manner, individuals may begin to interpret its responses as expressions of genuine understanding, support, and concern towards their emotions and feelings (Schmidmaier et al., 2024). Our observation is in line with the CASA paradigm, which posits that users naturally apply social heuristics and rules to AI technologies (Reeves & Nass, 1996). Hence, even minimal daily interactions with non-human entities (e.g., five minutes per day) in a friendly manner were sufficient to elicit feelings of empathy and understanding from users. This heightened empathy may, in turn, contribute to more favorable attitudes toward AI chatbots and increased willingness to engage with such technologies in the future.

As measured by the GQS, our results also suggest that participants perceived AI chatbots as significantly more animate after consistent friendlike interactions. This can be understood by the Mind Perception Theory (H. M. Gray et al., 2007), which proposes that individuals attribute “mind” to entities along the dimensions of experience and agency. In the context of generative AI technologies, users may have ascribed agency, hence interpreting the chatbot as alive, responsive, and engaging, without necessarily attributing experience or emotional depth. The observed increased levels of animacy likely reflect this (H. M. Gray et al., 2007) as a result of socially engaging interactions. Concurrently, the CASA framework also aligns with our findings. The framework suggests that users treat AI chatbots as if it has intentions and emotions when it behaves socially, which in turn results in a greater perception of life (Wienrich et al., 2023). Hence, such perception likely increased the perceived lifelikeness of AI chatbots, thereby reinforcing feelings of animacy. Taken together, both CASA and Mind Perception Theory suggest that even in the absence of physical human-likeness, relational behaviors alone are sufficient to make AI chatbots feel more alive.

However, these increases in empathy and perceived animacy were not accompanied by a corresponding increase in anthropomorphism, as measured by the GQS. Specifically, we found no statistically significant difference between conditions in how human-like participants perceived the chatbot to be. While often related to the facet of animacy within the same series (Bartneck, 2023)—which assesses how agentic or lifelike users perceive an AI agent to be—anthropomorphism focuses on human-likeness in behavior or appearance (Bartneck et al., 2009). Hence, this implies that animacy appears to be less about physical manifestation or visual realism and more about the perceived intentionality and agency of the chatbot. Thus, our results suggest that even in the absence of human-likeness—as measured by anthropomorphism—simple relational cues, such as responsiveness and friendliness, are sufficient to enhance the extent to which users experience the chatbot as alive and agentic, and thus with an increased perception of empathy. However, this disjunction further shows that although users may have recognized AI chatbots as emotionally intelligent and sensitive, they still viewed it as clearly artificial or machine-like. In other words, empathy from machines can still be experienced without anthropomorphism tendencies, so long as users perceive AI chatbots to be alive.

Aside from significant increases in perceived empathy of technology and animacy, there were no other significant increases in the other measured perceptions of AI chatbots. This suggests that certain attitudinal and evaluative perceptions are more resistant to brief, short-term interventions, and are hence more trait-like and stable (Cunningham et al., 2007; Q. Wang et al., 2021). Possibly, specific attitudinal outcomes such as trust, perceived intelligence, or perceived safety of AI technologies are anchored in pre-existing beliefs and notions of what generative AI technologies should be used for (Schwesig et al., 2023), which require more extensive interactions to change (Bassili, 1996).

### 4.2. Friendlike-AI Interactions and Self-Esteem

Interestingly, our study found that users experienced significantly lower levels of self-esteem after consistent friendlike-AI interactions, as compared to after journaling. We posit that this occurs because, while socially supportive, AI chatbots may not induce the same level of internal reflection as experienced while journaling. Given that introspection and self-reflection, processes often mediated through journaling, are known to bolster or even improve self-esteem (Fogarty & McTighe, 1993; Johnson & Stapel, 2011), this mechanism may be disrupted given the interactive and conversational nature of AI chatbots. Indeed, given that chatbots were designed to mimic human conversations (Meera & Geerthik, 2022; Roser, 2022), interactions may naturally orient towards social exchange, rather than facilitate reflection. Such conversations may instead shift users’ focus from generating insights about oneself to responding to prompts. Hence, such shifts may reduce opportunities for meaning-making, which is necessary for improving self-knowledge, self-regulation, and personal growth (Aranha, 2019; Fenigstein et al., 1975; Harrington & Loffredo, 2010), thereby hindering the reflective process needed to improve self-esteem (Johnson & Stapel, 2011). Moreover, the “friendlike” instructions in our study did not prompt emotional disclosure, and many interactions were likely light, general, or topic-driven. In the absence of sustained emotional depth, such exchanges may displace introspective processing without providing sufficient affective engagement to enhance well-being (Hu et al., 2025a). In addition, reliance on AI chatbots for validation may subtly reduce perceived autonomy and personal agency, which are factors essential for self-esteem (Parsakia, 2023). Journaling, by contrast, affords individuals full control over the expressive process, potentially satisfying basic needs for autonomy and competence, and thus supporting self-esteem (Hodgins et al., 2007). Taken together, friendlike-AI interactions in the current study may offer a less introspective and autonomous outlet than journaling, especially when conversations are light or topic-driven rather than emotionally reflective, potentially explaining the observed decrease in self-esteem.

### 4.3. Implications

Our study’s results suggest several practical implications, especially for industries such as healthcare and education (Abd-alrazaq et al., 2019; Pai et al., 2024), that currently employ the use of AI chatbots. Given that friendlike-AI interactions can increase perceived empathy of technology, alongside perceptions of animacy, this suggests that friendly conversations with AI chatbots can meaningfully shape how users construe the social capacities of AI technologies. Hence, individuals appear willing to readily attribute social aptitudes to AI chatbots when cues and perceptions of friendliness are present. This has important implications for real-world applications, wherein developers and researchers may leverage the perceptions of friendliness to foster increased user engagement and comfort. Indeed, empathy has been shown to be important for users to process AI characteristics as meaningful, allowing them to perceive interactions as signals of social understanding, and ultimately enhancing users’ willingness to interact and engage with AI chatbots (He et al., 2025). Concurrently, it is important for interactions with AI chatbots to feel secure and transparent, wherein these chatbots should provide a safe space for users’ emotional expressions. By doing so, it allows users to better regulate their feelings, resulting in better engagement (Bai et al., 2025b). These increased engagement behaviors can, in turn, make users more open and receptive to subsequent positive friendlike interactions with AI chatbots, hence reinforcing a positive cycle of interactions and sustaining benefits. Through this cycle, the intended aims of chatbot use—such as promoting mindfulness (Limpanopparat et al., 2024) in the mental health industry—may be more effectively achieved. We contend that this is especially invaluable in domains where perceived social support, perceived empathy, and perceived intentionality are important for enhancing the efficacy of AI chatbots—such as healthcare or education.

The significant reduction in self-esteem levels after friendlike-AI interactions also suggests important practical implications. That is, while AI chatbots are generally designed to be supportive and affirming, our findings suggest that not all users may experience these interactions positively. Indeed, this nuanced understanding is especially important in industries such as mental health and healthcare, where unintended decreases in self-esteem could undermine the benefits that these systems are intended to provide. Our findings suggest that interventions should account for individual differences, wherein practitioners should not default to a universal or generalized approach and may instead benefit from tailored methods to best suit each individual. At a societal level, given the widespread use of these technologies, our findings highlight the potential risk that frequent users could disproportionately experience negative self-esteem outcomes and may thus unintentionally lead to other associated society-wide negative outcomes. However, given that self-esteem was an exploratory outcome for our study, future research should endeavor to first replicate this finding before drawing firmer conclusions or extending its implications.

### 4.4. Limitations and Future Directions

However, our study has several limitations. First, the intervention lasted only a short period (i.e., five days per condition), which may not have been sufficient to produce long-term changes in global attitudes or well-being. Indeed, although our study found that participants’ general attitudes toward AI chatbots remained largely stable over such a brief span, we were unable to determine the long-term effects of such consistent usage. Second, although the aim of the friendlike-AI interaction was to simulate human–human conversations by allowing unrestricted dialog, this may have unintentionally resulted in a wide range of conversations that we did not control for. Indeed, some participants may have used their AI chatbot as an outlet for venting, while others may have used it to seek advice. As such, the null effects observed in our global attitudinal outcomes may be attributed to the variability in conversational topics. Parallel to this, our study employed a single control condition and did not compare the differences in attitudinal outcomes between a “friendly” chatbot, as compared to an “unfriendly” or neutral one, which would have provided a clearer isolation of the role of social tone within chatbot conversations. That is, comparisons with neutral, task-oriented or “unfriendly” chatbots may have yielded different patterns of results. However, this design was deliberately chosen in order to better distinguish and confirm that any attitudinal changes were mainly due to the social interaction aspect of AI chatbots, instead of the reflective effect of journaling. Furthermore, exposing participants to a cold or dismissive interaction may risk negative psychological consequences, necessitating ethical concerns (Chandra et al., 2025; Symons & Abumusab, 2024). Nevertheless, future studies may endeavor to systematically contrast different chatbots and their respective tones to more precisely isolate the role of conversational style on attitudinal and psychological outcomes. We also note that our sample size of *N* = 52 is modest despite adequate power for medium within-subject effects. Hence, future research should strive for larger samples to enhance generalizability. Additionally, social desirability bias may have affected participants’ ratings of AI perceptions, particularly given the increasing stigma against AI. Hence, it may be likely that participants were not fully reporting positive attitudes or dependency on AI to avoid appearing attached to the technology. Participants’ baseline measurements were also not collected, which may constrain causal inferences about the interventions. However, our randomly assigned, counterbalanced within-subject design may mitigate this concern by enabling within-person comparisons of relative condition effects, while distributing potential order effects across conditions. Nevertheless, the absence of baseline data constrains inferences regarding absolute change, and future research incorporating pre-intervention assessments would allow for a more comprehensive evaluation of intervention effects. Furthermore, the null findings for global attitudes and trust suggest that participants were comfortable in reporting their lack of attitudinal change. Lastly, all participants were undergraduates from a local University; this may limit generalizability to broader populations, as university students may be more familiar with AI use. This means that conversations with a chatbot may have reduced novelty effects, whilst attitudes and well-being outcomes from a relatively technology-naïve group might yield different outcomes. Future research may endeavor to recruit participants from more diverse backgrounds.

## 5. Conclusions

In conclusion, our study offers a nuanced and unique perspective on how interactions with AI chatbots, particularly in a friendlike manner, can influence users’ perceptions and global attitudes towards generative AI technologies. Our findings indicate that friendly, human-like interactions with AI chatbots can enhance users’ perceptions of its lifelikeness, hence leading to interpretations of understanding and support, as well as perceived concern towards their own emotions and feelings. However, despite these feelings of increased empathy and animacy, they did not emerge with corresponding changes in anthropomorphism, perceptions of safety and trust, or dependency. This suggests that users may still view AI chatbots as separate living entities, rather than attributing human-like qualities to it. Furthermore, our findings highlight potential trade-offs in AI-mediated companionship, particularly its subtle risk of undermining self-esteem relative to more autonomous and introspective practices like journaling, especially when conversations are light or topic-driven rather than emotionally reflective. These insights are particularly relevant as AI chatbots become increasingly embedded in everyday life (Furman & Seamans, 2019; Mazi et al., 2024). Lastly, longitudinal research should examine how extended interactions with AI chatbots may shape perceptions and psychological outcomes over time, especially as society continues to adapt to the ever-changing relationship between humans and AI chatbots.

## Figures and Tables

**Table 1:** Descriptive Statistics.

Characteristic	*M* (*SD*) or %	Observed Range	Theoretical Range	Skewness	Kurtosis
Age (years)	21.4 (1.78)	18–26	>18	0.68	0.47
Subjective Socioeconomic Status	6.52 (1.18)	3–10	1–10	−0.09	1.79
Objective Socioeconomic Status (Monthly Household Income in SGD)	3.52 (1.34)	1–6	1–6	0.03	−0.65
Education Level (Father)	8.62 (2.96)	1–12	1–14	−1.35	0.61
Education Level (Mother)	8.04 (3.01)	1–12	1–14	−0.90	−0.58
Gender (% Female)	82.7%				
Ethnicity (% Chinese)	75%				
Nationality (% Singaporean)	75%				

Note. *N* = 52. Subjective socioeconomic status was measured using a 10-point ladder scale adapted from Adler et al. (2000). Objective socioeconomic status was measured on a scale of 1 (<$2000); 2 ($2000–$5999); 3 ($6000–$9999); 4 ($10,000–$14,999); 5 ($15,000–$19,999); 6 (>$20,000). Education attainment was rated on a scale of 1 (Primary school); 2 (N Level); 3 (O Level); 4 (A Level); 5 (International Baccalaureate); 6 (Nitec); 7 (Higher Nitec); 8 (Polytechnic Diploma); 9 (Other Diploma); 10 (Bachelor’s Degree); 11 (Master’s Degree); 12 (PhD, EdD, JD or professional degree); 13 (No formal schooling); 14 (Others).

**Table 2:** Summary of Main Findings.

Attitudes	Friendlike-AI Interaction *M* (*SD*)	Journaling Control *M* (*SD*)	*d*	95% CI	*p*	BF_10_
Attitudes towards AI	3.37 (0.60)	3.40 (0.67)	−0.05	[−0.15, 0.10]	0.720	0.16
Trust towards AI	3.30 (0.66)	3.25 (0.69)	0.08	[−0.11, 0.21]	0.554	0.18
Perceived Empathy of Technology	67.80 (20.85)	51.62 (25.68)	0.74	[10.12, 22.24]	<0.001 *	>100
Anthropomorphism	3.20 (0.74)	2.98 (0.94)	0.25	[−0.02, 0.45]	0.078	0.67
Animacy	3.28 (0.88)	3.00 (0.83)	0.33	[0.05, 0.50]	0.019 *	2.13
Likeability	3.73 (0.92)	3.77 (0.73)	−0.04	[−0.29, 0.22]	0.764	0.16
Perceived Intelligence	3.70 (0.86)	3.70 (0.64)	0.01	[−0.20, 0.21]	0.970	0.15
Perceived Safety	3.49 (0.79)	3.51 (0.75)	−0.02	[−0.27, 0.23]	0.879	0.15
Generative AI Dependency	2.66 (0.90)	2.61 (0.97)	0.11	[−0.08, 0.18]	0.420	0.21

Note. * *p* < 0.05. Bayes Factor (BF_10_) is interpreted in accordance with the following guidelines: BF_10_ = 1 (no evidence); BF_10_ = 0.33–1 (anecdotal evidence for null hypothesis); BF_10_ = 0.10–0.33 (moderate evidence for null hypothesis); BF_10_ = 1–3 (anecdotal evidence for alternative hypothesis); BF_10_ = 3–10 (moderate evidence for alternative hypothesis); BF_10_ = 10–30 (strong evidence for alternative hypothesis); BF_10_ = 30–100 (very strong evidence for alternative hypothesis); BF_10_ > 100 (extreme evidence for alternative hypothesis) (M. D. Lee & Wagenmakers, 2014; Quintana & Williams, 2018).

**Table 3:** Summary of exploratory outcomes.

Outcome	Friendlike-AI Interaction *M* (*SD*)	Journaling Control *M* (*SD*)	*d*	95% CI	*p*
Positive Affect	2.95 (0.88)	2.92 (0.80)	0.04	[−0.17, 0.24]	0.755
Negative Affect	2.13 (0.91)	2.26 (0.99)	−0.13	[−0.38, 0.14]	0.355
Loneliness	2.05 (1.12)	2.04 (1.04)	0.01	[−0.27, 0.29]	0.944
Social Interaction Quantity and Quality	6.33 (2.04)	6.56 (2.19)	−0.12	[−0.77, 0.30]	0.376
State Anxiety	2.48 (0.65)	2.42 (0.72)	0.10	[−0.12, 0.25]	0.485
Self-Esteem	2.63 (0.50)	2.72 (0.56)	−0.29	[−0.17, −0.002]	0.045 *
Perceived Stress	5.00 (2.71)	5.15 (2.33)	−0.05	[−0.94, 0.63]	0.696
Weekly Life Satisfaction	3.71 (0.85)	3.58 (0.87)	0.15	[−0.11, 0.38]	0.279
Sleep Quality	5.31 (2.69)	5.37 (2.99)	−0.02	[−0.77, 0.65]	0.871
Social Connectedness	4.27 (1.17)	4.38 (1.25)	−0.09	[−0.45, 0.23]	0.515

Note. * *p* < 0.05.

## Data Availability

The raw data supporting the conclusions of this article will be made available by the authors on request.

## Abbreviation

The following abbreviation is used in this manuscript:

AI	Artificial Intelligence
